# Treatment outcomes of radiotherapy with concurrent weekly cisplatin in older patients with locally advanced head and neck squamous cell carcinoma

**DOI:** 10.1007/s12672-023-00844-7

**Published:** 2023-12-08

**Authors:** Yusuke Uchinami, Koichi Yasuda, Satoshi Kano, Manami Otsuka, Seijiro Hamada, Takayoshi Suzuki, Nayuta Tsushima, Shuhei Takahashi, Yoshihiro Fujita, Tomohiko Miyazaki, Hajime Higaki, Jun Taguchi, Yasushi Shimizu, Tomohiro Sakashita, Akihiro Homma, Hidefumi Aoyama

**Affiliations:** 1 Department of Radiation Oncology, Hokkaido University Faculty of Medicine and Graduate School of Medicine, North 15 West 7, Kita-Ku, Sapporo, Hokkaido 060-8638 Japan; 2 Department of Radiation Oncology, Hokkaido University Hospital, North 14 West 5, Kita-Ku, Sapporo, Hokkaido 060-8648 Japan; 3 Department of Otolaryngology-Head and Neck Surgery, Hokkaido University Faculty of Medicine and Graduate School of Medicine, North 15 West 7, Kita-Ku, Sapporo, Hokkaido 060-8638 Japan; 4 Department of Medical Oncology, Hokkaido University Faculty of Medicine and Graduate School of Medicine, North 15 West 7, Kita-Ku, Sapporo, Hokkaido 060-8638 Japan; 5 Department of Otolaryngology-Head and Neck Surgery, Kushiro City General Hospital, Syunkodai 1-12, Kushiro, Hokkaido 085-0822 Japan

**Keywords:** Chemoradiotherapy, Head and neck cancer, Older patients, Treatment outcomes, Weekly cisplatin

## Abstract

**Background:**

Tri-weekly cisplatin and radiotherapy (CDDP + RT) is a standard of care for locally advanced head and neck squamous cell carcinoma (LA-HNSCC) but is sometimes challenging to complete in older patients. Weekly CDDP + RT has shown mild toxicity compared to tri-weekly CDDP + RT for LA-HNSCC and is a promising option for older adults. We aimed to report the treatment outcomes and prognostic factors in patients with LA-HNSCC treated with weekly CDDP + RT.

**Methods:**

We analyzed patients aged ≥ 70 years who started weekly CDDP + RT for LA-HNSCC between July 2006 and October 2022. LA-HNSCC includes cancer in the oropharynx, hypopharynx, or larynx with a clinical stage of 3 or 4 without distant metastases based on the Union for International Cancer Control staging system 8th edition. The radiation dose of 70 Gy was delivered in 35 fractions by 3-dimensional conformal radiotherapy, intensity-modulated radiotherapy, or proton beam therapy. The primary endpoint was the 3-year overall survival (OS), and the secondary endpoints were the 3-year progression-free survival (PFS) and 3-year cause-specific survival (CSS). The Kaplan–Meier method was used to calculate survival rates, and the log-rank test was used to evaluate statistical significance. A Cox proportional hazards model was used for the multivariate analysis of prognostic factors.

**Results:**

The median age of the 49 patients was 72 (range: 70–78) years. The median CDDP dose was 200 (40–280) mg/ m^2^, and 47 patients completed scheduled radiotherapy. Forty-eight patients (98.0%) had a performance status of ≥ 1 at the initial visit. The 3-year OS, PFS, and CSS were 80.9% (95% confidence interval [CI]: 64.8–90.7), 58.9% (95%CI: 42.7–73.3), and 85.0% (95%CI: 68.7–93.4), respectively. In the multivariate analysis, the cumulative CDDP dose (< 200 or ≥ 200 mg/m^2^) was a significant factor for OS (hazard ratio: 0.29 [95% CI 0.08–0.97], p = 0.044). There was one case of early mortality. Grade 3 or higher late adverse events were observed in four patients (8.2%).

**Conclusions:**

Weekly CDDP + RT in older patients led to good survival outcomes with an acceptable rate of adverse events. CDDP should be administered at a dose of at least 200 mg/m^2^ in older patients.

*Trial registration* Retrospectively registered

## Background

The standard of care for the non-surgical treatment of locally advanced head and neck squamous cell carcinoma (LA-HNSCC) is chemoradiotherapy (CRT), a combination of 70 Gy radiotherapy and concurrent cisplatin (CDDP) 100 mg/m^2^ given every 3 weeks for a total of three cycles (3-weekly CDDP + RT) [1, 2]. However, compared with younger patients, older patients often have impaired general conditions or organ functions, and the 3-weekly CDDP + RT is sometimes challenging to complete [3]. Therefore, radical treatments with reduced intensity should be used in such cases.

Non-standard CRT regimens for older patients include weekly CDDP + RT, weekly carboplatin (CBDCA) plus RT, cetuximab plus RT, and RT alone [4]. To date, no randomized trial has compared the optimal regimen for these patients. Of these, weekly CDDP + RT with doses of CDDP 30–40 mg/m^2^ is considered the standard of care for locally advanced nasopharyngeal carcinoma [5] and postoperative high-risk patients with recurrent head and neck squamous cell carcinoma (HNSCC) [6]. Furthermore, weekly CDDP (40 mg/m^2^) was the most commonly used CRT regimen in older patients with HNSCC in German-speaking countries or global surveys [4, 7]. Thus, weekly CDDP + RT may be a candidate for older patients with difficulty with the standard 3-weekly CDDP + RT.

To the best of our knowledge, the treatment results with weekly CDDP + RT in older patients are minimal. We believe that it is necessary to report the outcomes to investigate optimal treatments in the future. This study aimed to report the survival outcomes of older patients with LA-HNSCC treated with weekly CDDP + RT.

## Methods

### Patients

The Ethics Review Board of our institution approved this study (IRB-022-0028). Written informed consent for treatment was obtained from all enrolled patients. We reviewed the medical records of patients who started radical CRT for LA-HNSCC between July 2006 and October 2022. The inclusion criteria were as follows; aged ≥ 70 years, CRT using weekly CDDP + RT, and cancer in the oropharynx, hypopharynx, or larynx with a clinical stage of 3 or 4 without distant metastases based on the Union for International Cancer Control (UICC) staging system (8th edition). Finally, 49 patients were included (Table 1). Patients were deemed refractory to standard 3-weekly CDDP + RT at a multidisciplinary conference attended by all medical professionals involved in the practice. Although there were no absolute criteria for the indication of chemotherapy, we considered the following factors: the patient’s general condition, organ function, and complications in each case. The enrolled older patients predominantly had an Eastern Cooperative Oncology Group (ECOG) performance status (PS) of ≥ 1 at the initial visit. Patients who underwent lymph node dissection prior to CRT and those who switched to weekly CBDCA therapy during treatment were also included in the analysis.

**Table 1:** Patient background data

Sex	
Male	43 (87.8%)
Female	6 (12.2%)
Median age	72 years (range: 70–78)
ECOG performance status	
0	1 (2.0%)
1	43 (87.8%)
2	4 (8.2%)
3	1 (2.0)
Primary tumor site	
Oropharynx	18 (36.7%), p16 positive: 1^a^
Hypopharynx	30 (61.2%)
Larynx	1 (2.0%)
T stage (UICC 8th)	
1	1 (2.0%)
2	23 (46.9%)
3	16 (32.7%)
4	9 (18.4%)
N stage (UICC 8th)	
0	9 (18.4%)
1	7 (14.2%)
2	30 (61.2%)
3	3 (6.1%)
Stage (UICC 8th)	
III	12 (24.5%)
IV	37 (75.5%)
Median CDDP dose (range)	200 mg/m^2^ (range: 40–280)
Median number of CDDP cycles	5 (range: 1–7)
Median radiation dose	70 Gy (range: 42–71)
Radiotherapy modality	
3DCRT	17 (34.7%)
IMRT	30 (61.2%)
Proton	2 (4.1%)
Neck dissection prior to CRT	2 (4.1%)

All patients were restaged according to the UICC 8th edition classification

*3DCRT* 3-dimensional conformal radiotherapy, *CDDP* cisplatin, *CRT* chemoradiotherapy, *ECOG* Eastern Cooperative Oncology Group, *HPV* human papillomavirus, *IMRT* intensity-modulated radiotherapy, *UICC* Union for International Cancer Control

^a^In all, nine patients were examined

### Contouring

If the patient had no allergies or other contraindications to the procedure, contrast-enhanced computed tomography (CT) was used for the planning CT. Enhanced or unenhanced magnetic resonance imaging (MRI) was used to assess the extent of the primary tumor or lymph node metastasis. The general contour procedure was as follows: the gross tumor volume (GTV) was contoured as the primary tumor, and lymph node involvement was determined using available imaging modalities, including CT, MRI, and positron emission tomography (PET). Clinical target volume 1 (CTV1) was created with a 5 mm margin added to the GTV. CTV1 was modified for areas of overlap with the air, bone, or outside the body. CTV2 was generated as a high-risk region with a potential tumor but without CTV1. CTV3 was also generated as a moderate- or low-risk region that included the entire neck, except for CTV1 and CTV2. For X-ray radiotherapy, planning target volume 1 (PTV1) was generated by adding a margin of 3–5 mm to CTV1. PTV2 was generated by adding a margin of 3–5 mm to CTV2, excluding PTV1 for more strict dose delivery to PTV2. Similarly, PTV3 was generated as CTV3 with a 3–5 mm margin, excluding both PTV1 and PTV2.

### Radiotherapy

The planned dose prescription was 70 Gy in 35 fractions for all study patients. In 3-dimensional conformal radiotherapy, 44–46 Gy was prescribed to the whole neck region, including the GTV, followed by a boost of 24–26 Gy using an X-ray or electron beam to the tumor and lymph node metastases. In intensity-modulated radiotherapy (IMRT), doses were prescribed for at least 95% of the PTV (PTV D95). In IMRT using the simultaneous integrated boost method, 70 Gy was prescribed to PTV1; 56–63 Gy and 50.4–56 Gy were prescribed to PTV2 and PTV3, respectively. In IMRT using the sequential boost method, 44–46 Gy was prescribed to all regions of PTV1, PTV2, and PTV3, then 24–26 Gy was prescribed to the reduced region, including the original GTV. In proton beam therapy using the intensity-modulated proton beam therapy (IMPT) technique, doses similar to those used in IMRT were prescribed for 99% volume of each CTV, assuming a relative biological effectiveness of 1.1. Robust optimization was used to prescribe the assumption of setup uncertainties of 3 mm and range uncertainties of 3.5%.

In photon radiotherapy, XiO (Elekta AB, Stockholm, Sweden), Pinnacle^3^ (Philips, Amsterdam, The Netherlands), or RayStation (RaySearch Laboratories AB, Stockholm, Sweden) were used as treatment planning systems. For the linear accelerator (LINAC), radiotherapy with a 6- or 10-mega voltage X-ray was delivered using Varian CL2300 (Varian Medical Systems, Palo Alto, CA), MHCL-15SPLINAC (Mitsubishi Electronics Co., Ltd., Tokyo), Varian CLINIC iX (Varian Medical Systems), or TrueBeam (Varian Medical Systems). In proton beam therapy with PROBEAT-RT (Hitachi, Ltd., Tokyo, Japan), VQA (Hitachi) was used for treatment planning.

### Chemotherapy

All patients received at least one cycle of concurrent chemotherapy during CRT. CDDP was administered weekly at 40 mg/m^2^ in 45 patients (91.8%). Other regimens administered 34, 30, and 20 mg/m^2^ of weekly CDDP. The cisplatin dose was adjusted based on the age and general condition of the patients. In three patients, the weekly cisplatin regimen was changed to a weekly carboplatin regimen. In two of them, the replacement was due to deterioration in renal function during CRT, while in the third patient, it was due to difficulty with hydration prior to CDDP administration. In the multivariate analysis, the cut-off dose of CDDP was defined as 200 mg/m^2^, according to previous reports [8–10].

### Follow-up

After CRT completion, patients were evaluated every 1–3 months during the first year, 3–4 months during the second and third years, and every 6–12 months after that, depending on the patient’s status. A laryngoscopy was performed at each follow-up visit. Computed tomography or magnetic resonance imaging studies were performed every 3 months during the first year and at most follow-up visits thereafter. Adverse events were assessed for grade 3 or higher hematologic and non-hematologic toxicities according to the Common Terminology Criteria for adverse events version 5.0 (CTCAE v5.0).

### Statistical analysis

The primary endpoint of the study was the 3-year overall survival (OS). The secondary endpoints were the 3-year progression-free survival (PFS) and 3-year cause-specific survival (CSS). The OS, PFS, and CSS with a 95% confidence interval (95% CI) were calculated from the first day of CRT. OS was calculated until the last follow-up day or the date of death. PFS was calculated until the day of recurrence, death, or the last follow-up day. For CSS, deaths due to causes other than LA-HNSCC were excluded. The Kaplan–Meier method was used to calculate survival rates, and the log-rank test was used to evaluate statistical significance. A Cox proportional hazards model was used for the multivariate analysis of prognostic factors. These analyses were performed using JMP Pro version 16 (SAS, Cary, NC, USA), and statistical significance was set at p < 0.05.

## Results

### Patient characteristics

The median age of the 49 patients was 72 years (range: 70–78 years), of which 12 (24.5%) were 75 years or older. Forty-three patients (87.8%) had an ECOG PS of 1. Because one patient had a PS of 0 but was relatively old (74 years), weekly CDDP + RT was selected for this patient. In addition, two patients underwent neck dissection prior to CRT. Forty patients (81.6%) received ≥ 4 cycles of CDDP, and 17 (34.7%) received ≥ 6 cycles.

Forty-seven patients (95.9%) completed their scheduled radiotherapy. The dose prescription was modified during CRT in two patients (71 Gy in 33 fractions or 66 Gy in 33 fractions) to account for the biological effects. The 71 Gy case was due to concerns about prolonged treatment duration during many calendar holidays. The 66 Gy case was due to a mid-treatment dosimetric evaluation that revealed a slightly excessive dose administration because of rapid tumor shrinkage. The treatment was discontinued in two patients (68 Gy in 34 fractions or 42 Gy in 21 fractions) due to adverse events. One discontinued at 68 Gy due to pneumonia and another discontinued at 42 Gy due to acute appendicitis.

Forty-five patients (91.8%) were administered weekly CDDP at 40 mg/m^2^; the administration in other patients was at 20–34 mg/m^2^ considering age and general condition prior to treatment. In three patients, the chemotherapy regimen was switched to weekly CBDCA during the chemotherapy cycle due to worsened renal function or increased urination frequency.

### Survival

**Fig. 1:**
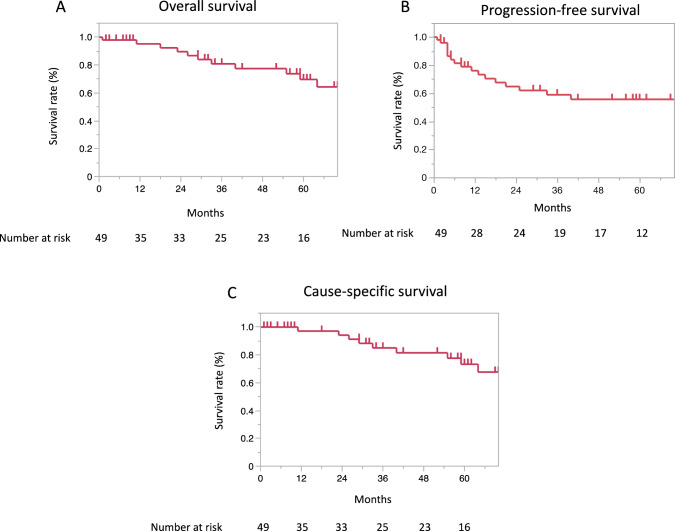
Graphs showing the overall survival (**A**), progression-free survival (**B**), and cause-specific survival (**C**) rates regarding all patients included in the study

With a median follow-up period of 36 months (range: 1–156), the 3-year and 5-year OS rates were 80.9% (95% CI 64.8–90.7) and 69.7% (51.6–83.2), respectively (Fig. 1A). The 3-year and 5-year PFS rates were 58.9% (95%CI: 42.7–73.3) and 55.6% (39.4-70.7), respectively, and those for the CSS rates were 85.0% (95%CI: 68.7–93.4) and 73.3% (54.5–86.3), respectively (Fig. 1B, 1C).

**Fig. 2:**
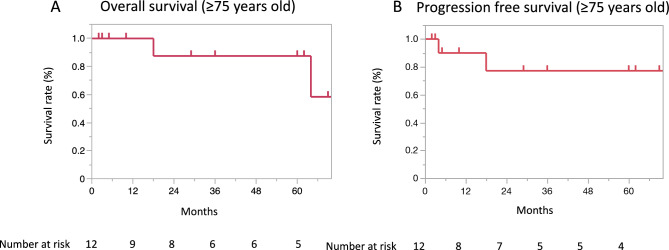
Graphs showing the overall survival (**A**) and progression-free survival (**B**) rates in the analysis limited to 12 patients aged ≥ 75 years

In an analysis limited to 12 patients aged ≥75 years, the median follow-up period was 32.5 months (range:2-77). The 3-year and 5-year OS rates were the same, 87.5% (95% CI 46.3–98.3), and the 3- and 5-year PFS rates were also the same, 77.1% (95% CI 40.5–94.4) (Fig. 2A, B).

In the univariate analysis of OS, age and cumulative dose of CDDP were considered to be potential prognostic factors (Table 2). Concerning age, the patients were divided into two groups according to a median age of 72 years. On the multivariate analysis for all patients, the cumulative CDDP dose (< 200 or ≥ 200 mg/m^2^) was a significant factor for better OS (hazard ratio: 0.29 [95% CI 0.08–0.97], p = 0.044).

**Table 2:** Univariate and multivariate analyses

	n	Univariate		Multivariate	
		3-year OS (%)	p-value	HR (95% CI)	p-value
Sex					
Male	43	84.2%	0.785		
Female	6	60.0%			
Age					
≤ 72 years	25	71.5	0.101	Reference	
≥ 73 years	24	89.5		0.32 (0.09–1.13)	0.076
ECOG PS					
0–1	44	78.4	0.897		
2–3	5	100			
Primary tumor site					
Oropharynx	18	83.3	0.848		
Hypopharynx/larynx	31	79.5			
Stage (UICC 8th)					
III	12	88.9	0.433		
IV	37	78.6			
Cumulative CDDP dose (mg/m^2^)					
< 200	21	73.2	0.055	Reference	
≥ 200	28	86.1		0.29 (0.08–0.97)	0.044
Radiotherapy modality					
3DCRT	17	100	0.272		
IMRT/proton	32	68.5			

*3DCRT* 3-dimensional conformal radiotherapy, *95% CI* 95% confidence interval, *CDDP* cisplatin, *ECOG* Eastern Cooperative Oncology Group, *HR* hazard ratio, *IMRT* intensity-modulated radiotherapy, *OS* overall survival, *PS* performance status

### Adverse events

The most common hematologic adverse event (≥ grade 3) was leukopenia in 16 patients (32.7%) (Table 3). Among the non-hematologic adverse events, ≥ grade 3 radiation-induced mucositis was observed in approximately half of the patients (51.0%). One patient died early during treatment (2.0%). He developed acute appendicitis at 42 Gy and three cycles of CDDP, which led to fatal gastrointestinal perforation. The association between acute appendicitis and CRT is uncertain, but myelosuppression caused by cisplatin may have had an effect. In cases where long-term follow-up was possible, grade 3 late adverse events were observed in four patients (Table 3). In these patients, soft tissue necrosis in the neck and jaw necrosis improved with surgical management.

**Table 3:** Acute and late adverse events of ≥ grade 3

Adverse event	Grade 3–4
Acute	
(Hematologic)	
Leukopenia	16 (32.7%)
Neutropenia	11 (22.4%)
Anemia	5 (10.2%)
Thrombocytopenia	1 (2.0%)
(Non-hematologic)	
Mucositis	25 (51.0%)
Radiation dermatitis	6 (12.2%)
Dysphagia	8 (16.3%)
AST/ALT increased	1 (2.0%)
Creatinine increased	0
Late	
Neck soft tissue necrosis	2 (4.1%)
Jaw necrosis	1 (2.0%)
Dysphagia	1 (2.0%)

*AST* aspartate transaminase, *ALT* alanine transaminase

## Discussion

This study reports the results of older patients with LA-HNSCC who were treated with weekly CDDP + RT. Of the 49 patients who started CRT, 47 (95.9%) completed the scheduled radiotherapy. The 3-year and 5-year overall survival (OS) rates were 80.9% and 69.7%, respectively. Multivariate analysis showed that the cumulative CDDP dose (< 200 or ≥ 200 mg/m^2^) was a prognostic factor for OS. We believe that these data will be important when considering optimal treatment for older patients.

This is the study reporting results of weekly CDDP + RT limited to older patients, although a few studies that included patients aged ≥ 70 years have been carried out. Ghosh et al. reported the results of weekly CDDP + RT in a prospective randomized controlled trial of LA-HNSCC [11]. They analyzed 65 patients with a median age of 55 years who underwent CRT and reported a 5-year OS rate of 56% and a 5-year PFS rate of 39%. In another retrospective series that included older adults, the 3-year OS was reported to be around 63.0–86.8% [12–15]. In our study, which included only patients aged ≥ 70 years, the 3-year OS rate was 80.9%. Furthermore, the 3-year OS rate was 87.5%, even when restricted to patients aged 75 years or older. Most of the results described above are from retrospective analyses, and there may be significant differences in patient backgrounds. However, our results of weekly CDDP + RT that are limited to older patients are comparable to those that include younger patients and may be a promising treatment option for older patients with LA-HNSCC.

The completion of scheduled CRT is essential for better OS in older patients. In the present study, 47 out of the 49 patients completed their scheduled radiotherapy, and 28 received CDDP at a dose of ≥ 200 mg/m^2^, the dose of which was considered potentially crucial for better OS on multivariate analysis. A previous study also reported that a total dose of approximately 200 mg/m^2^ CDDP, regardless of the schedule of administration, may be effective in achieving a positive outcome [16]. However, in general, older patients do not tolerate CDDP administration as do younger patients. Omata et al. analyzed 146 patients treated with high-dose CDDP (80 mg/m^2^ every 3 weeks) for HNSCC, classifying patients by age 70 years (< 70 or ≥ 70 years) [3]. The results showed that the PS and cumulative dose of CDDP were significantly lower in the ≥ 70-year-old group. Thus, chemotherapy regimens that allow older patients to complete as much of the scheduled CRT as possible while minimizing adverse events are needed.

In terms of adverse events, weekly CDDP + RT is a promising option for the older cohort. The advantage of weekly CDDP + RT is that it is less toxic than 3-weekly CDDP + RT because of the reduced (30–40 mg/m^2^) single dose [6, 17]. In a phase 3 trial comparing weekly CDDP (40 mg/m^2^) + RT to 3-weekly CDDP + RT in the postoperative setting, Kiyota et al. reported that the weekly CDDP + RT arm was non-inferior in OS [6]. The study also showed that adverse events were less common in the weekly CDDP + RT arm. Although their study results were from a younger population (the median age of the entire population was 62 years) and involved adjuvant CRT, the fewer adverse events of weekly CDDP + RT may be more advantageous to older patients.

The present study focused on patients aged ≥ 70 years, and the overall incidence of acute adverse events was similar to that reported in previous retrospective studies of weekly CDDP + RT [12, 14, 15, 18]. It should be noted that from a radiotherapy perspective, more recently published studies may include more cases treated with IMRT or proton beam therapy, and have an advantage regarding adverse events. In contrast, in a prospective study of weekly CDDP + RT in patients with HNSCC with a median age of 55 years, the incidences of ≥ grade 3 acute hematological adverse events, dermatitis, and mucositis were 5.3%, 15%, and 23%, respectively [11]. The radiotherapy technique used in this prospective study was the more classical 2-dimensional radiotherapy, but it still tended to have a lower incidence of adverse events than that in our study. Moreover, the present study showed that 25 patients (51.0%) had ≥ grade 3 mucositis, suggesting that supportive care during CRT may be more important for older patients.

As this was a single-center retrospective study, there were several limitations. The number of cases was limited to approximately 50, and the observational period needed to have been increased in some cases. There are also differences in radiotherapy modalities, from 3DCRT to proton beam therapy, depending on the treatment time. Most previous studies comparing 3DCRT and IMRT in HNSCC did not show a difference in OS or local control rates. However, they reported a significantly lower number of adverse events in patients treated with IMRT [19, 20]. In addition, simulation studies of IMRT versus proton beam therapy have suggested that there may be an advantage in terms of acute-adverse events [21]. In contrast, the strength of our study is that the treatment regimen during the study period was generally consistent with 70 Gy in 35 fractions and weekly CDDP at 40 mg/m^2^. We believe that our results with weekly CDDP + RT will be helpful in future clinical trials in older adults with LA-HNSCC.

## Conclusions

The results of weekly CDDP + RT for older patients with LA-HNSCC in this study were comparable to those of previous studies that included younger patients. The standard of care for older patients with difficulty with the standard 3-weekly CDDP + RT has not been established, and further prospective comparative trials are warranted.

## Data Availability

Research data are stored in an institutional repository and will be shared upon request to the corresponding author.

## Abbreviations

3DCRT: 3-Dimensional conformal radiotherapy
CBDCA: Carboplatin
CDDP: Cisplatin
CI: Confidence interval
CRT: Chemoradiotherapy
CSS: Cause-specific survival
CTCAE: Common terminology criteria for adverse events
ECOG: Eastern Cooperative Oncology Group
IMRT: Intensity-modulated radiotherapy
LA-HNSCC: Locally advanced head and neck squamous cell carcinoma
OS: Overall survival
PFS: Progression-free survival
PS: Performance status
UICC: Union for International Cancer Control
