# Correction: Treatment outcomes of radiotherapy with concurrent weekly cisplatin in older patients with locally advanced head and neck squamous cell carcinoma

**DOI:** 10.1007/s12672-024-00868-7

**Published:** 2024-01-25

**Authors:** Yusuke Uchinami, Koichi Yasuda, Satoshi Kano, Manami Otsuka, Seijiro Hamada, Takayoshi Suzuki, Nayuta Tsushima, Shuhei Takahashi, Yoshihiro Fujita, Tomohiko Miyazaki, Hajime Higaki, Jun Taguchi, Yasushi Shimizu, Tomohiro Sakashita, Akihiro Homma, Hidefumi Aoyama

**Affiliations:** 1 Department of Radiation Oncology, Hokkaido University Faculty of Medicine and Graduate School of Medicine, North 15 West 7, Kita‑Ku, Sapporo, Hokkaido 060‑8638 Japan; 2 Department of Radiation Oncology, Hokkaido University Hospital, North 14 West 5, Kita‑Ku, Sapporo, Hokkaido 060‑8648 Japan; 3 Department of Otolaryngology‑Head and Neck Surgery, Hokkaido University Faculty of Medicine and Graduate School of Medicine, North 15 West 7, Kita‑Ku, Sapporo, Hokkaido 060‑8638 Japan; 4 Department of Medical Oncology, Hokkaido University Faculty of Medicine and Graduate School of Medicine, North 15 West 7, Kita‑Ku, Sapporo, Hokkaido 060‑8638 Japan; 5 Department of Otolaryngology‑Head and Neck Surgery, Kushiro City General Hospital, Syunkodai 1‑12, Kushiro, Hokkaido 085‑0822 Japan

**Correction: Discover Oncology (2023) 14:226** 10.1007/s12672-023-00844-7

After publication an error was found by the authors in the progression-free survival (PFS) analysis. In the original analysis, deaths were not included in the progression-free survival (PFS) analysis as events, as noted in the Methods section. The errors caused by this are listed in this correction article. This analytical error does not affect the interpretation and conclusions of the original article [1].

**Abstract**

Incorrect

- The 3-year OS, PFS, and CSS were 80.9% (95% confidence interval [CI]: 64.8–90.7), 68.3% (95% CI 51.8–81.2), and 85.0% (95% CI 68.7–93.4), respectively.

Correct

- The 3-year OS, PFS, and CSS were 80.9% (95% confidence interval [CI]: 64.8–90.7), **58.9**% (95% CI: **42.7–73.3**), and 85.0% (95% CI: 68.7–93.4), respectively.

**Results**

Incorrect

- With a median follow-up period of 36 months (range: 1–156), the 3-year and 5-year OS rates were 80.9% (95% CI 64.8–90.7) and 69.7% (51.6–83.2), respectively (Fig. 1A). The 3-year and 5-year PFS rates were 68.3% (95% CI 51.8–81.2) and 68.3% (51.8–81.2), respectively, and those for the CSS rates were 85.0% (95% CI 68.7–93.4) and 73.3% (54.5–86.3), respectively (Fig. 1B, C).
- In an analysis limited to 12 patients aged ≥ 75 years, the median follow-up period was 32.5 months (range: 2–77). The 3-year and 5-year OS rates were the same, 87.5% (95% CI 46.3–98.3), and the 3- and 5-year PFS rates were also the same, 53.3% (95% CI 53.3–98.6) (Fig. 2A, B).

Correct

- With a median follow-up period of 36 months (range: 1–156), the 3-year and 5-year OS rates were 80.9% (95% CI 64.8–90.7) and 69.7% (51.6–83.2), respectively (Fig. 1A). The 3-year and 5-year PFS rates were **58.9**% (95% CI: **42.7–73.3**) and **55.6**% (**39.4–70.7**), respectively, and those for the CSS rates were 85.0% (95% CI: 68.7-93.4) and 73.3% (54.5-86.3), respectively (Fig. 1B, C).
- In an analysis limited to 12 patients aged ≥75 years, the median follow-up period was 32.5 months (range:2-77). The 3-year and 5-year OS rates were the same, 87.5% (95% CI 46.3–98.3), and the 3- and 5-year PFS rates were also the same, **77.1**% (95% CI **40.5–94.4**) (Fig. 2A, B).

Incorrect

- The median age of the 49 patients was 72 years (range: 70–78) years.

Correct

- The median age of the 49 patients was 72 years (range: 70–78 years).

Incorrect figure 1b
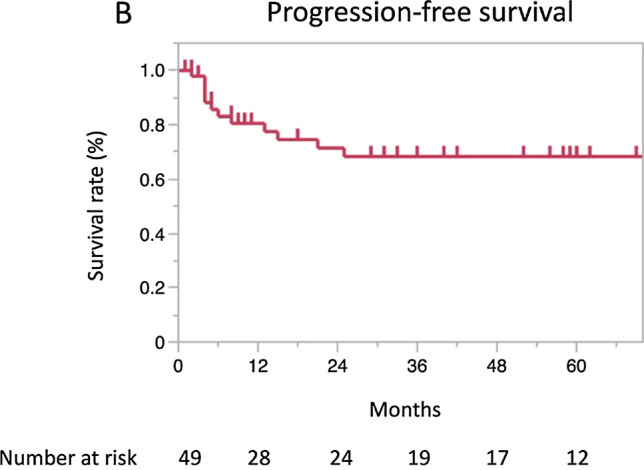


Correct figure 1b
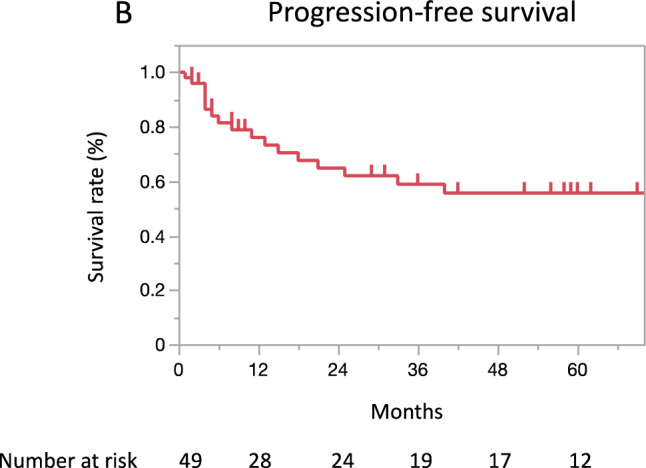


Incorrect figure 2b
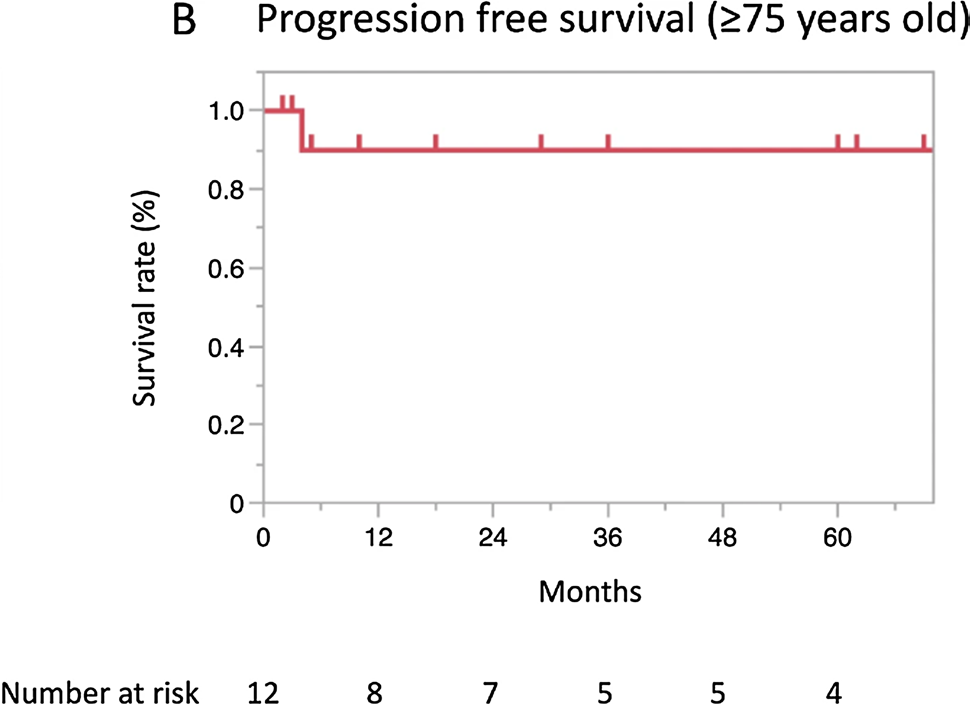


Correct figure 2b
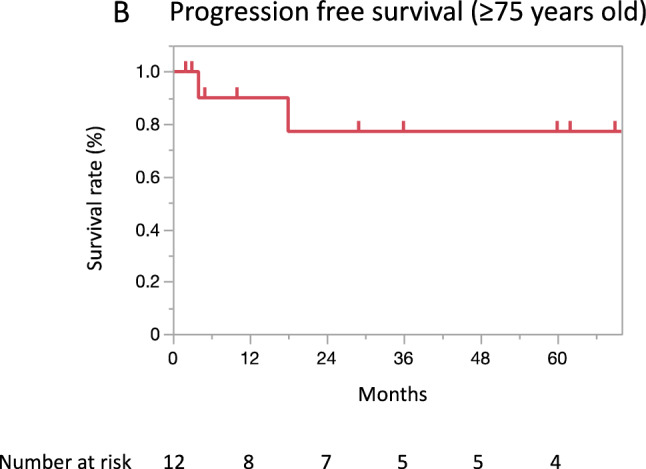


## References

[1] Uchinami Y, Yasuda K, Kano S, Otsuka M, Hamada S, Suzuki T, Tsushima N, Takahashi S, Fujita Y, Miyazaki T, Higaki H, Taguchi J, Shimizu Y, Sakashita T, Homma A, Aoyama H (2023). Treatment outcomes of radiotherapy with concurrent weekly cisplatin in older patients with locally advanced head and neck squamous cell carcinoma. Discover Oncol.

